# Rainbow trout (*Oncorhynchus mykiss*) secretory component binds to commensal bacteria and pathogens

**DOI:** 10.1038/srep41753

**Published:** 2017-02-02

**Authors:** Cecelia Kelly, Fumio Takizawa, J. Oriol Sunyer, Irene Salinas

**Affiliations:** 1 Center for Evolutionary and Theoretical Immunology, Department of Biology, University of New Mexico, Albuquerque, NM 87131-0001, USA; 2 Pathobiology Department, School of Veterinary Medicine, University of Pennsylvania, Philadelphia, USA

## Abstract

Commensal bacteria co-exist on the mucosal surfaces of all vertebrates. The host’s mucosal immune system must tolerate commensals while fighting pathogens. One of the mechanisms used by the mucosal immune system to maintain homeostasis is the secretion of immunoglobulins (Igs) across epithelial barriers, which is achieved via the polymeric immunoglobulin receptor (pIgR). Rainbow trout pIgR is known to transport IgT and IgM across epithelia. However, other biological functions for trout pIgR or trout secretory component (tSC) remain unknown. This study investigates the interaction of tSC with commensal bacteria, pathogenic bacteria and a fungal pathogen. Our results show that the majority of trout skin and gut bacteria are coated *in vivo* by tSC. *In vitro*, tSC present in mucus coats trout commensal isolates such as *Microbacterium sp., Staphylococcus warneri, Flectobacillus major, Arthrobacter stackebrantii,* and *Flavobacterium sp*. and the pathogens *Vibrio anguillarum* and *Edwardsiella ictaluri* with coating levels ranging from 8% to 70%. Moreover, we found that the majority of tSC is in free form in trout mucus and free tSC is able to directly bind bacteria. We propose that binding of free SC to commensal bacteria is a key and conserved mechanism for maintenance of microbial communities in vertebrate mucosal surfaces.

Vertebrate animals draw many physiological benefits from the symbiotic communities that live in their mucosal surfaces. Living together with complex microbial communities requires a number of exquisite mechanisms to keep these microorganisms in check. For instance, the bacteria found in the gut lumen of mammals are coated by secretory immunoglobulins (sIgs) in a process known as immune exclusion^1^. In mammals, sIgs arrive at the mucus layer via transcytosis across the epithelium by the polymeric immunoglobulin receptor (pIgR), an Fc receptor expressed on the basal surfaces of epithelial cells^2^. Once pIgR transports the sIg across the epithelium, pIgR is cleaved and a portion remains associated with the sIg polymer, known as the secretory component (SC)^3^.

Teleost fish also have a pIgR that is analogous to human pIgR^4^,^5^. Trout pIgR has some conserved features when compared to mammalian pIgR, but also important differences. Trout pIgR only has two of the five immunoglobulin-like domains present in mammalian pIgR. Domains 1 and 5 of the trout SC (tSC) are homologous to domains 1 and 5 of the human SC (D1 and D5)^6^. D1 interacts with both Fc regions and the J-chain of polymeric Igs in humans. D5 further stabilizes the interaction via a disulphide bond between a cysteine residue in D5 and the Cα2 region of one of the dimeric IgA^7^. In teleosts, the cysteine required for this disulphide bond is absent in D5^5^, and there is no known J-chain present in trout sIgs for D1 to associate with^8^,^9^. Additionally, three loops structurally similar to complementary determining regions in Igs, which are involved in the association between human sIgA and SC, are not conserved in trout D1^7^. Currently, the only known function for teleost pIgR is the transport of IgM and IgT into mucosal secretions^5^,^10^,^11^.

sIgs and pIgR are molecules that have co-evolved alongside the commensal microbiota of the host, resulting in the development of advanced interactions between both parties. In mammals, the sIg-SC complex is known to limit access of microbes to the epithelium and help to control commensal populations. This is achieved through antigen specific, Fab-mediated binding and innate Fab-independent (glycan mediated) binding, both present in the sIg molecule^12^,^13^,^14^,^15^. SC is also heavily glycosylated, which allows for additional interactions with microorganisms including direct microbial binding in its free form^15^,^16^,^17^. Similar to mammalian sIgs, both IgT and IgM coat commensal bacteria present in the skin, gut, gill and nasal mucus of rainbow trout^5^,^10^,^11^,^18^. However, the interactions between teleost pIgR and microorganisms have never been investigated.

Apart from the role of human SC as a scavenger molecule, few studies have also shown that certain pathogens such as *Streptococcus pneumoniae*, have evolved mechanisms of hijacking the pIgR transport system in order to adhere to and invade the host epithelium^19^,^20^. Thus, pIgR/SC not only determines the health status of mammalian hosts by shaping commensal communities, but also can serve as an avenue for certain pathogens to invade the epithelium. It is therefore plausible that teleost pIgR/SC is exploited by a number of aquatic pathogens such as bacteria and fungi as a mechanism for host invasion.

To date, no studies have been conducted on the interactions between rainbow trout commensal bacteria and pathogens and pIgR/SC. We hypothesize that the ability of SC to interact with bacterial proteins is conserved in rainbow trout, and that these interactions may play a major role in shaping the commensal communities present in rainbow trout mucosal surfaces. It is also possible that harmful fish pathogens have evolved mechanisms of binding pIgR to gain access to the host epithelium. Our results indicate that SC binds to several members of the normal rainbow trout microbial community, as well as fish pathogens. This suggests that the functional advantages as well as vulnerabilities associated with human SC may be conserved in rainbow trout.

## Results

### tSC coats trout gut and skin commensals *in vivo*

**Figure 1:**
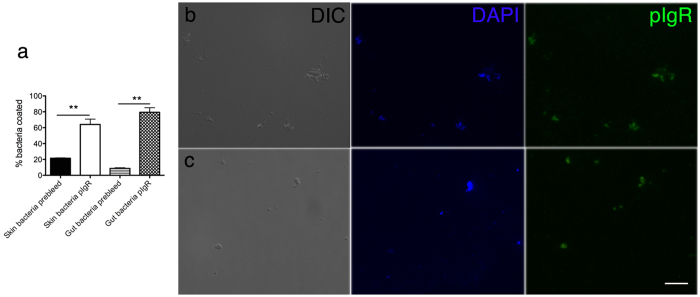
Skin and gut commensals are coated *in vivo* with tSC. Skin and gut associated bacteria were isolated as explained elsewhere^5^,^10^,^11^ and labeled with rabbit anti-trout pIgR antibodies or the prebleed control followed by a secondary Cy2-donkey anti-rabbit IgG. (**a**) Mean percentage ± SEM of gut and skin bacteria coated with tSC in control rainbow trout (N = 3). (**b**) Immunofluorescence microscopy images of trout gut bacteria labeled with anti-trout pIgR from left to right: differential interference contrast (DIC), DNA stain DAPI (blue), and anti-pIgR (Cy2, green). (**c**) Immunofluorescence microscopy images of trout skin bacteria labeled with anti-trout pIgR from left to right: differential interference contrast (DIC), DNA stain DAPI (blue), and anti-pIgR (Cy2, green). **Indicate statistically significant differences (p < 0.01) by student T-test. Results are representative of one experiment.

In order to show that rainbow trout gut and skin commensals are coated *in vivo* with pIgR/SC, we examined freshly isolated trout skin and gut bacteria using immunofluorescence microscopy. We observed that 64% of bacteria isolated from the skin were coated with pIgR, while 79% of gut bacteria were coated with pIgR, compared to background coating levels with the prebleed antibody of 20% and 9% in skin and gut, respectively (Fig. 1a–c).

### Bacterial acquisition of tSC from skin mucus *in vitro*

Trout skin mucus harvested from hatchery trout was filtered to remove pre-existing bacteria and incubated with lab-cultured commensal and pathogenic bacterial strains to assess binding of tSC to surface epitopes of a variety of trout-associated commensal and pathogenic bacteria. Coating of cultured bacterial strains by tSC present in the collected mucus was then measured using flow cytometry. Table 1 shows the bacterial strains used, their characteristics and sources. We found that all six bacterial species tested were coated to varying degrees by tSC (Table 2 and Supplementary Fig. S1). The mean percentage coating of each bacteria species from highest to lowest was: *Flavobacterium sp.* (70.9%) > *V. anguillarum* (33%) > *F. major* (26.2%) > *S. warneri* (15.6%) > *Microbacterium sp.* (14.7%) > *E. ictaluri* (8.3%) (Table 2).

### F. major tSC coating levels are lower than sIgT coating levels

**Figure 2:**
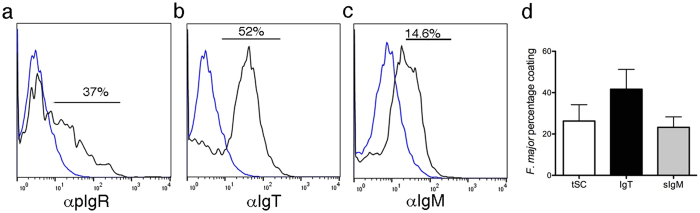
Flow cytometry studies showing acquisition of tSC, sIgT or sIgM by *F. major in vitro*. *F. major* cells were labeled with BacLight red and incubated with either skin mucus or serum overnight. After washing, bacteria were labeled with rabbit anti-pIgR, rabbit anti-IgT or mouse anti-IgM antibodies or the corresponding isotype controls followed by FITC donkey anti-rabbit IgG or FITC-donkey anti-mouse IgG and measured by flow cytometry. (**a**) Overlay histogram of *F. major* coating by tSC. (**b**) Overlay histogram of *F. major* coating by sIgT. (**c**) Overlay histogram of *F. major* coating by sIgM. BacLight^+^ cells were gated prior to analysis. Blue line: isotype control. Black line: antibody stained samples. Gate was placed based on the corresponding serum negative control. (**d**) Mean percentage ± SE coating of *F. major* with tSCs, sIgT and sIgM. Results are representative of two independent experiments (N = 3).

We next sought to compare tSC, sIgT and sIgM coating levels using *F. major* as a model commensal strain. We found that following overnight incubation with skin mucus, 26.2 ± 13.7% of all *F. major* cells were coated with tSC, 41.6 ± 16.4% were coated with IgT and 14.6 ± 8.8% were coated by IgM (Fig. 2a–d).

### F. major maximal coating with tSC precedes maximal coating with IgT

**Figure 3:**
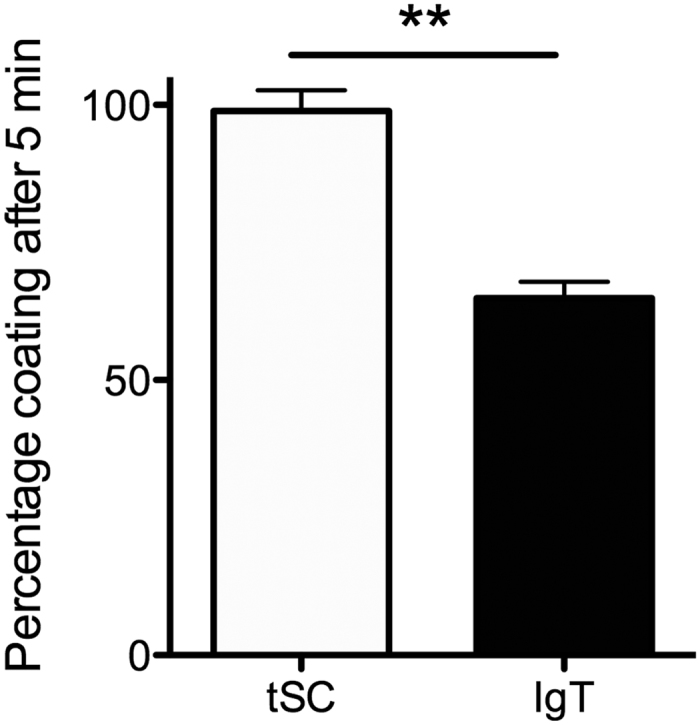
*F. major* maximal tSC coating takes place before maximal sIgT coating. *F. major* cells were labeled with BacLight red and incubated with either skin mucus or serum for 5 min or overnight. After washing, bacteria were labeled with rabbit anti-pIgR or anti-IgT antibodies or the corresponding pre-bleed controls followed by FITC donkey anti-rabbit IgG antibody and measured by flow cytometry. Mean percentage tSC and sIgT coating of *F. major* BacLight^+^ cells after 5 min compared to overnight (maximal) coating levels (N = 3). Results are representative of three independent experiments. **Indicate statistically significant differences (p < 0.01) by student T-test.

In order to better understand the kinetics of commensal bacteria coating by tSC and sIgs, we performed very short incubations (5 min) with *F. major* and skin mucus and compared them to overnight incubations, our standard protocol to assess binding of microorganisms to mucus proteins. As shown in Fig. 3~99% of tSC coating occurs within 5 min whereas only ~69% of IgT coating occurs in the same period of time.

### The majority of tSC is found in free form in trout mucosal secretions

**Figure 4:**
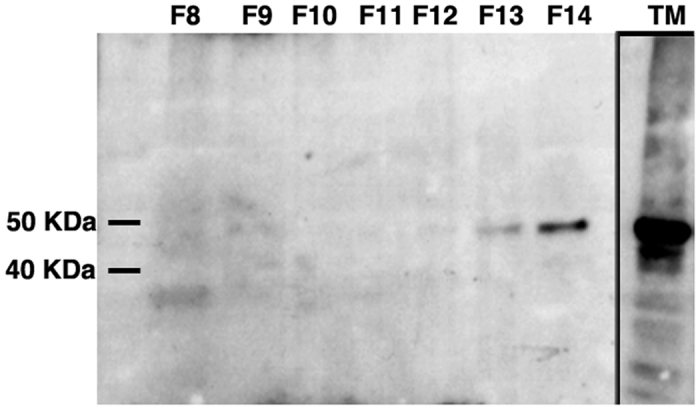
The majority of tSC is found in free form in rainbow trout mucosal secretions. Immunoblot of rainbow trout gill mucus following gel filtration. Fractions 8–14 (F8–14) are shown. F8–9 contains trout sIg^5^,^10^,^11^ (not shown) and very low levels of tSC could be detected. F14 corresponding to tSC in its free form contains the majority of the SC present in the sample. TM: total trout gill mucus before fractionation. Results are representative of two different mucus pools.

In order to know whether tSC is found in association with sIgs or in free form we fractionated trout gill mucus by gel filtration followed by immunoblotting of the fractions with anti-trout pIgR. As shown in Fig. 4, the majority of tSC eluted in fraction 14 ml, and not fractions 8–9 ml, where trout polymeric sIgs elute^5^,^10^,^11^.

### Free tSC directly binds commensal and pathogenic bacteria *in vitro*

Flow cytometry analysis using purified tSC and the different commensal and pathogenic bacterial strains revealed that free tSC is able to directly bind to bacterial cell walls. Based on the percentage of coating the order from higher to lower coating levels was as follows: *Flavobacterium sp.* (42%) > *E. ictaluri* (8.3%) > *V. anguillarum* (5.4%) > *F. major* (2.4%) > *S. warneri* (1.8%) > *Microbacterium sp.* (1.1%) (Table 3).

### tSC from total trout mucus but not free purified free tSC binds the fungal pathogen M. hiemalis

**Figure 5:**
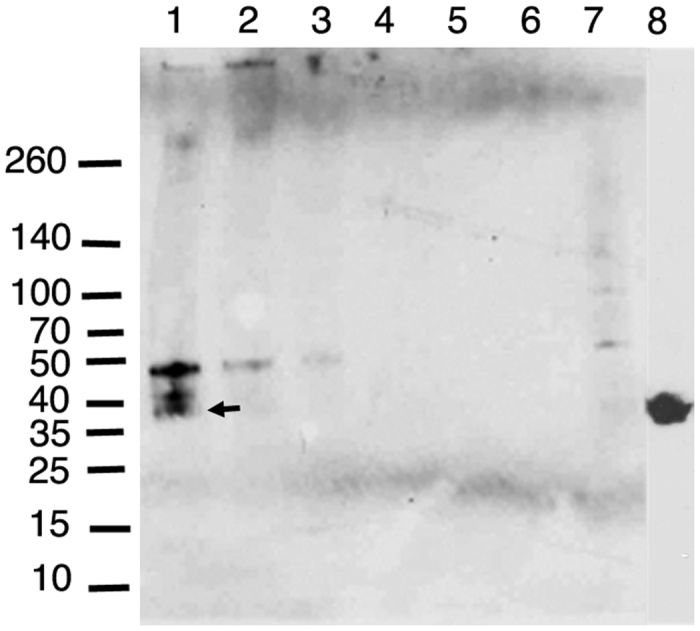
Pull-down assay of trout pIgR/SC from gut mucus using *M. hiemalis*. Lane 1. Undiluted *M. hiemalis* culture, Lane 2. *M. hiemalis* 1:10 dilution, Lane 3. *M. hiemalis* 1:100 dilution, Lane 4. Undiluted *M. hiemalis* culture incubated with trout serum, Lane 5. *M. hiemalis* 1:10 dilution incubated with trout serum, Lane 6. *M. hiemalis* 1:100 dilution incubated with trout serum. Lane 7. Undiluted *M. hiemalis* culture not incubated with mucus or serum. Lane 8. Gut mucus sample used in the pull-down assay showing the presence of trout pIgR (38 KDa band) as previously reported^5^. Lane 8 was cropped from a separate western blot and pasted onto the pull-down results. Results are representative of two different experiments. Arrow indicates the 38 KDa band corresponding to trout gut pIgR/SC.

In order to study the interactions between tSC present in trout mucus and the fungal pathogen *M. hiemalis* we performed a pull-down assay. We successfully pulled down trout pIgR from gut mucus using three different concentrations of *M. hiemalis*. We pulled down three different bands when *M. hiemalis* was incubated with trout gut mucus. The largest of the three bands was also faintly present in the lane where *M. hiemalis* alone was run (Fig. 5). This band did not have the expected size of gut pIgR (∼38 KDa). A second band of (∼41 KDa) was also pulled down and was not considered pIgR since it did not have the expected molecular weight. The lowest molecular weight band pulled down by incubating *M. hiemalis* with trout gut mucus had the expected molecular weight of trout gut pIgR (Fig. 5, arrow). The negative control consisting of trout serum, which does not contain pIgR, did not result in the precipitation of any bands (Fig. 5). Purified tSC was also used to attempt to pull down *M. hiemalis*. Unlike the pull down assay using total trout gut mucus, no *M. hiemalis* was pulled down using free tSC (not shown).

### Proposed models of tSC interactions with microbial surfaces

**Figure 6:**
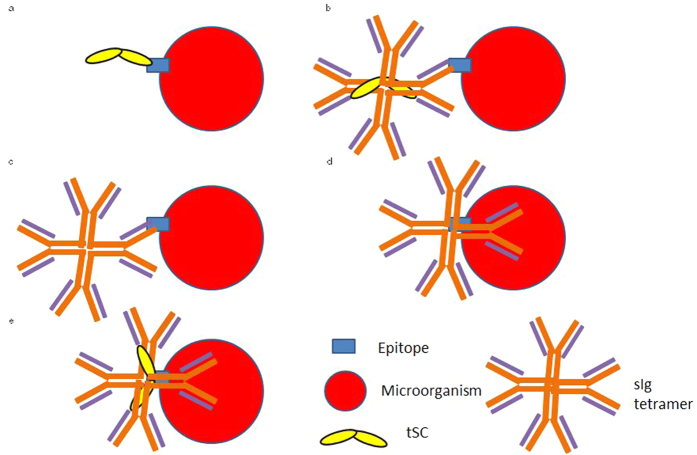
Proposed model of tSC interaction with microbial surfaces. Schematic representation of the proposed mechanisms of trout sIgs and tSC interactions with microbial surface epitopes. (**a**) Free tSC binds to a bacterial epitope in a glycan-mediated manner. (**b**) The Fab region of sIg (IgT or IgM) binds specifically to a bacterial epitope, and tSC remains associated with the sIg complex. (**c**) The Fab region of sIg (IgT or IgM) binds specifically to a bacterial epitope, and tSC is not associated with the sIg complex. (**d**) sIgs bind bacterial epitopes in a glycan mediated manner without tSC still associated with the complex. (**e**) sIgs bind bacterial epitopes in a glycan mediated manner with tSC present in the complex. Scenarios (**b** and **e**) may include simultaneous binding of sIgs and tSC to different microorganisms or epitopes within one microorganism.

Since tSC exists both as a free glycoprotein and in association with the sIgT/IgM complex in the mucus, and sIgs may bind microorganisms specifically via their antigen binding sites or nonspecifically via glycan-mediated binding, we propose a model which shows every potential mechanism of binding between microorganisms and pIgR/SC-sIgs in trout (Fig. 6a–e). The scenario depicted in Fig. 6a is likely to be the most common in rainbow trout secretions upon first encounter of a new bacterial species and not prominent in the case of Gram-positive bacteria. Scenarios depicted in Fig. 6b and c appear to be the most common in the case of *F. major*. Additionally, even if not considered in this figure, epitopes involved in glycan-mediated binding to pIgR or sIgs may be different to those recognized specifically by the antigen-binding sites of sIgs. Finally, other mechanisms of interaction different from antigen binding site binding and glycan-mediated binding may exist.

## Discussion

The mucosal immune system of teleosts protects the fish by maintaining a mucosal barrier that keeps pathogens at bay and commensal populations in check. Previous studies show that trout sIgs coat commensal bacteria on the skin and in the gut^5^,^10^, and that these sIgs are transported across the epithelium by trout pIgR^5^. However, to date, the interactions between trout pIgR/SC and microorganisms had not been investigated.

Here we show for the first time that tSC coats skin and gut bacteria *in vivo*. The levels of coating exceed IgT and IgM coating levels of trout commensals previously reported in gut and skin^5^,^10^. Commensal bacteria isolated from the rainbow trout gut have been previously reported to be ~48% coated with IgT and ~24% coated with IgM, while ~38% and ~12% of skin commensals were coated with IgT and IgM respectively^5^,^10^. The latter suggested that free tSC coats commensal bacteria. These *in vivo* findings were supported by the experiments performed using purified free tSC. In humans, up to 50% of pIgR traffics to the apical surface and is released as free SC^21^.

In order to identify species-specific interactions between trout pIgR/SC and bacteria, we tested a number of pure microbial cultures. Our results show that tSC in conjunction with sIgs or in free form interacts with a wide variety of commensal and pathogenic bacteria to different degrees depending on the species. Out of all the isolates tested, *Flavobacterium sp.* had the highest levels of tSC coating in total mucosal secretions. Similar results were obtained when free tSC was used. *Flavobacteriaceae* are ubiquitous members of the rainbow trout microbiome^22^,^23^,^24^. Thus, it appears that SC coating is a pivotal mechanism by which the host is able to maintain *Flavobacterium sp.* communities on its mucosal surfaces.

We observed that tSC combined with sIgs or in its free form is able to interact with both Gram-positive and Gram–negative bacteria (as well as fungi). Interestingly, we observed the lowest amount of free tSC coating in *S. warneri* and *Microbacterium sp.,* both Gram-positive bacteria. Human SC binds to bacterial epitopes in a glycan-mediated manner^17^,^25^. Specifically, previous studies have shown that N-linked glycans on human SC play a vital role in the association of sIgA with Gram-positive cell walls^25^, which are made up of a thick layer of peptidoglycan and contain teichoic acids. Overall, our results indicate that free tSC does not bind Gram-positive bacteria as efficiently as human SC does, likely due to key differences in the N-linked glycans present in teleost and mammalian SC.

*F. major* is the main bacterial species found in the gill and skin microbiota of rainbow trout^22^. Thus, we selected this species to make comparative studies aiming to evaluate *de novo* coating of bacteria with tSC, IgT and IgM. In the presence of total mucus, the level of IgT coating exceeded that of tSC coating. Moreover, coating levels in the presence of free tSC were significantly lower than those detected using total mucus. The latter indicates that in the case of *F. major*, the primary mechanism of interaction with trout mucosal secretions is binding to sIgs with or without SC attached to the complex. This result differs from previous human studies where no differences in sIgA and SC coating were detected using a number of bacterial strains^25^. Interestingly, short incubation experiments revealed that maximal tSC coating levels of *F. major* are achieved very rapidly *in vitro* whilst IgT coating levels continued to increase over time. Since *in vitro* growth conditions do not mimic *in vivo* conditions, translating these results to an *in vivo* scenario is complex. Our data nevertheless suggest that presence of tSC in the *F. major*-sIg complex may mediate further binding of additional sIg molecules to the microbial surface and that sIg binding may continue to occur under tSC saturation.

Recently, X-ray crystallography has been used to generate a three-dimensional model of the free form of human SC and teleost SC. This study revealed that mammalian SC evolved to adopt a compact, closed triangular structure, which opens upon ligand binding, whereas the two-domain teleost SC consists of tandem domains with an open elongated conformation^7^. Importantly, the same study showed that the structure of human SC changes when antibodies bind to it^7^. The interactions between tSC and bacterial surface proteins appear to work in conjunction with specific or non-specific binding between sIgT/IgM and bacterial epitopes. Supporting this notion, synergistic effects between SC and sIgs have been reported in mammals. For instance, in the case of murine dimeric IgA, carbohydrate residues in SC ensure the correct anchoring of the antibodies in the mucus layer^26^. Additionally, SC increases the efficiency of sIgA to clear bacterial pathogens in the mouse respiratory tract^26^. In this manner, similar to mammalian SC, tSC could further strengthen the association between sIgs and microbial epitopes, increase the efficiency of sIg function, increase the coating ability of sIgs or ensure the correct anchoring of sIgs in the mucus layer.

Interactions between pIgR/SC and bacteria do not always benefit the host. For instance, the *S. pneumoniae* colonizes human epithelial cells via pIgR^19^,^20^. Thus, the possibility that interactions between fish pathogens and tSC benefit the pathogen rather than the host cannot be ruled out. Our results show that tSC interacts with surface epitopes of the Gram-negative fish enteropathogen *E. ictaluri* and the mucosal fish pathogen *V. anguillarum. E. ictaluri* is an intracellular pathogen that causes enteric septicemia in channel catfish (*Ictalurus punctatus*) but it does not typically infect salmonids and has been shown to invade fish intestinal epithelial cells^27^. Interestingly, the coating levels here reported using total mucus and free tSC were very similar. Thus, as expected, no interactions between *E. ictaluri* and trout sIgs were present in our study and therefore all the *E. ictaluri* coating occurred via the free tSC mechanism. Thus, free SC may be responsible for adhesion of bacterial cells upon first encounter when the trout immune system has not generated specific antibodies against that particular species. *V. anguillarum* is known to enter the fish host via skin, gut and gill epithelial layers^28^,^29^. Our results may indicate that both *E. ictaluri* and *V. anguillarum* may take advantage of the pIgR system to invade the host’s epithelium, although further experiments need to be conducted in order to confirm this hypothesis.

Fungal cell walls are generally composed of chitin, glucans, mannans, and glycoproteins, which could potentially interact non-covalently with glycans or amino acid residues of SC. Additionally, fungal cell walls are also extensively modified with carbohydrate containing molecules, which are sensed by the innate immune system, and can activate inflammatory responses^30^. The present study provides the first evidence that not only bacteria but also fungi can adhere to the mucus layer via interactions with SC. We found, however, that unlike bacteria, the interaction between tSC and *M. hiemalis* requires the presence of sIgs. *Mucor sp.* is known to infect teleosts including rainbow trout^22^. Additionally, although yet unknown, it is likely that *Mucor sp.* as well as other fungi, are part of the normal microbiota of teleost fish. The presence of tSC in the trout sIg-fungi interaction could contribute to the formation and maintenance of fugal microbiomes, the exclusion of pathogenic fungus from entering the epithelium or the blocking of epitopes usually recognized by innate immune sensors to prevent innate immune activation.

In conclusion, here we show for the first time the *in vivo* and *in vitro* interactions between tSC and different commensals and pathogens in teleost fish. Despite obvious molecular differences between mammalian and teleost pIgR, the present study provides evidence of a conserved role for SC in maintaining microbial communities in vertebrate mucosal surfaces.

## Methods

### Animals and collection of mucus samples

Trout were obtained from Lisboa Springs, Pecos, New Mexico, and had a mean weight of 250 g. Skin mucus was scraped using a sterile glass slide and placed onto a sterile Petri dish. The whole gut was removed, placed on a sterile Petri dish, and opened longitudinally. The gut mucus was scraped from the lumen and one ml of ice-cold protease inhibitor buffer (1 × PBS, containing 1 × protease inhibitor cocktail (Roche), 0.5% bovine serum albumin (Sigma), 1 mM phenylmethylsulfonyl fluoride (Sigma); pH 7.2) was added to each sample. Gill mucus was collected as previously described^11^. Briefly, to obtain the mucus, blood in the gills was first removed by perfusion with PBS–heparin through the heart until the gills were completely blanched. Gill arches were excised and rinsed with PBS three times to remove the remaining blood. Thereafter, gills were incubated for 12 h at 4 °C, with occasional shaking in protease inhibitor buffer at a ratio of 1 g of gill tissue per ml of buffer.

Mucus suspensions were collected into Eppendorf tubes, vigorously vortexed and centrifuged sequentially at 40 g, 400 g, and 10,000 g in order to remove large particles, fish cells, and finally to pellet all bacteria. The resulting supernatant was harvested for *in vitro* experiments. To ensure no bacteria were present for flow cytometry experiments, mucus samples were filtered through a 0.45 μm filter (Milipore) and stored at −80 °C to prevent protein degradation until use. The presence of pIgR in every mucus sample was confirmed by western blot prior to use (Supplementary Fig. S2). As previously published^5^,^10^,^11^, pIgR in trout gut mucus had a molecular weight of ∼38 KDa whereas in skin and gill mucus, the size was ∼45 KDa.

All experimental protocols were approved by by the Institutional Animal Care and Use Committee (IACUC) at the University of New Mexico, protocol number 16-200384-MC. The methods in the present study were carried out in accordance with the relevant guidelines and regulations.

### Microorganisms

Commensal bacteria were isolated from healthy hatchery rainbow trout and cultured in the lab in Luria Bertani (LB) broth and LB agar plates. DNA from pure overnight cultures was extracted and strains were identified by sequencing of the 16S rDNA using the P46 forward primer and P943 reverse primers (P46 forward, 5′-GCYTAAYACATGCAAGTCG-3′, and P943 reverse, 5′-ACCGCTTGTGCGGGYCC-3′) as explained elsewhere^22^. The source, characteristics and accession numbers of all microorganisms used in this study are shown in Table 1.

For *in vitro* experiments, *S. warneri, Microbacterium sp., A. stackebrantii, V. anguillarum* and *Flavobacterium sp.* were grown overnight at 24 °C in LB broth. *E. ictaluri* was grown in brain heart infusion overnight at 27 °C and *F. major* was grown in ATCC *F. major* culture medium at 27 °C. *Mucor hiemalis* was isolated from sick rainbow trout from an Oregon trout facility, and identified by rDNA sequencing as explained elsewhere^22^. The fungus was cultured on R2A plates for three days at room temperature. To induce sporulation, *M. hiemalis* was cultured in 1% tryptone liquid media.

### SDS-PAGE and Western Blots

Ten μl of each trout mucus pool sample were mixed with 10 μl of Laemlli sample buffer (Bio-Rad) and were separated using 4–15% Mini-PROTEAN^®^ TGX™ precast gels (Bio-Rad) under non-reducing conditions and transferred onto PVDF membranes (Thermo Scientific). Membranes were blocked overnight in 5% non-fat milk (LabScientific) and then incubated for 1 h with primary antibody (1:1000 rabbit anti-trout pIgR), followed by 45 min with secondary antibody (1:2500 horseradish peroxidase-conjugated donkey anti-rabbit IgG, Jackson Immunoresearch). Membranes incubated with rabbit prebleed antibody were used as a negative control. After three washes, membranes were developed using Pierce^®^ ECL western blotting solution and exposed in darkroom to CL-X Posure™ film (Thermo) or scanned using Bio-Rad ChemiDoc™ XRS + with Image Lab™ Software.

### Purification of free tSC by Gel Filtration

Gill mucus diluted in PBS was applied to gel filtration to analyze the proportions of free tSC present and purify the free tSC fractions. Fractions containing pIgR were separated by gel filtration using a Pharmacia Superdex 200 column (GE healthcare). The column was previously equilibrated with cold PBS, and protein fractions were eluted at 0.5 ml/min with PBS using a fast protein liquid chromatography instrument with ÄKTApurifier systems (GE Healthcare). Protein elution was monitored by absorbance at 280 nm. Identification of free tSC in the eluted fractions was performed by western blot analysis using anti-pIgR polyclonal antibody as described above.

### Pull-down assay

*Mucor hiemalis* grown in 1% tryptone was washed once in 500 μl of PBS and the pellet was heavily vortexed until homogenized. The homogenized fungus sample was diluted 1:10 and 1:100 in PBS. The original homogenized fungal sample as well as the serial dilutions were incubated with 500 μl of trout gut mucus or purified tSC overnight at 4 °C under continuous rotation. Negative control samples consisted of the same fungus samples, but incubated with trout serum (1:500 diluted in PBS). The next day, each sample was spun down at 1000 g for 10 min, and the supernatant was removed. The pellet was washed three times in PBS, and the pellets were loaded onto SDS-PAGE gels and transferred to a PVDF membrane. Western blots for the detection of pIgR were performed as described above to detect the pulled down tSC.

### Flow cytometry

Overnight bacteria cultures of each strain were washed in PBS and stained with BacLight Red (Life Technologies) as per manufacturer’s instructions. After blocking with PBS containing 1% bovine serum albumin (BSA) on ice for 15 min, samples were incubated overnight at 4 °C with skin mucus containing 2 mM EDTA, serum diluted 1:500 in PBS containing 2 mM EDTA or purified free tSC. For *F. major* coating experiments, additional experiments were performed where bacteria were incubated for either 5 min or overnight. Bacterial samples were then washed three times with PBS containing 0.1% BSA. Bacteria were incubated with primary antibody (rabbit anti-trout pIgR, rabbit anti-trout IgT or mouse anti-trout IgM) for 1 h on ice, washed three times in PBS containing 0.1% BSA, and finally incubated with FITC-donkey anti-rabbit IgG secondary antibody or FITC-donkey anti-mouse IgG (both from Jackson Immunoresearch) for 45 min. Negative controls consisted of a rabbit prebleed or a mouse IgG1 isotype control as primary antibody.

After three washes in PBS, samples were resuspended in 200 μl of PBS and analyzed in a BD FACSCalibur flow cytometer. A total of 10,000 events were recorded. In order to quantify the % of bacteria coated with tSC, the BacLight positive population was first gated and the % of FITC^+^ bacteria present in this population was recorded.

### Microscopy

Bacterial pellets isolated from healthy rainbow trout (N = 3) gut and skin mucus obtained as explained elsewhere^5^,^10^. Bacterial suspensions were placed onto sterile microscope slides and were immediately blocked with T20 protein blocking solution (ThermoFisher) for 15 min. Slides were incubated with primary anti-trout pIgR antibody or the prebleed antibody as a negative control, followed by Cy2-conjugated donkey anti-rabbit secondary antibody (Jackson Immunoresearch). Slides were then stained with a solution containing DAPI (1 μg/mL) and Hoescht (2 μg/mL) DNA stain for 30 min, rinsed in tap water, and mounted with fluorescent mounting media. Slides were observed under a Nikon Ti fluorescent microscope, and images acquired with the NIS Advanced Research Software v.4. A total of ten images (x60) per sample were captured, and the numbers of Cy2^+^ and Cy2^−^ bacteria were counted in each image.

### Statistical analysis

Results are expressed as the mean ± standard error (SE). Data analysis was performed in GraphPad Prism version 5.0. Results were analyzed by unpaired T-test to identify statistically significant differences between groups. Statistically significant differences were considered when p < 0.05.

## Additional Information

**How to cite this article**: Kelly, C. *et al*. Rainbow trout (*Oncorhynchus mykiss*) secretory component binds to commensal bacteria and pathogens. *Sci. Rep.* **7**, 41753; doi: 10.1038/srep41753 (2017).

**Publisher's note:** Springer Nature remains neutral with regard to jurisdictional claims in published maps and institutional affiliations.

## Supplementary Material

Supplementary Information

## Figures and Tables

**Table 1:** List of the microorganisms used in the present study.

*Species*	Description	Source	Accession number	Reference
*Microbacterium sp.*	Gram-positive commensal bacteria	Healthy rainbow trout skin mucus	KX233500	This paper
*Staphylococcus warneri*	Gram-positive commensal bacteria	Healthy rainbow trout skin mucus	KX233499	31
*Flectobacillus major*	Gram-negative commensal bacteria	ATCC 29496	NA	NA
*Arthrobacter stackebrantii*	Gram-negative commensal bacteria	Healthy rainbow trout skin mucus	KR709316	22
*Flavobacterium sp.*	Gram-negative commensal bacteria	Healthy rainbow trout skin mucus	KX197203	This paper
*Edwarsiella ictaluri*	Gram-negative bacterial pathogen	J100 strain from infected channel catfish	NA	27
*Vibrio anguillarum*	Gram-negative bacterial pathogen	Provided by D. Milton	NA	31
*Mucor hiemalis*	Fungal pathogen	Gills of diseased rainbow trout	KR709320	22

**Table 2:** Bacterial acquisition of tSC from skin mucus *in vitro.*

Species	Mean % tSC coating with mucus ± SE	Mean % tSC coating with serum ± SE
*Microbacterium sp.*	14.7 ± 7.2	3.8 ± 0.2
*S. warneri*	15.6 ± 6.5	7.5 ± 6.6
*F. major*	26.2 ± 13.7	6.7 ± 0.5
*Flavobacterium sp.*	70.9 ± 14.4	2.7 ± 2.4
*E. ictaluri*	8.3 ± 1	1.6 ± 0.7
*V. anguillarum*	32.9 ± 17.5	7.5 ± 6.5

**Table 3:** Bacterial acquisition of purified free tSC *in vitro*.

Species	% tSC coating
*Microbacterium sp.*	1.1
*S. warneri*	1.8
*F. major*	2.4
*Flavobacterium sp.*	42
*E. ictaluri*	8.3
*V. anguillarum*	5.4
