# Supplementary material for: Rainbow trout (Oncorhynchus mykiss) secretory component binds to commensal bacteria and pathogens
Source: Sci Rep. 2017 Feb 2;7:41753. doi: 10.1038/srep41753 (PMC5288726; doi:10.1038/srep41753)

Rainbow trout (Oncorhynchus mykiss) secretory component binds commensal bacteria and pathogens

Cecelia Kelly[1] , Fumio Takizawa[2], J. Oriol Sunyer[2], Irene Salinas[1]*

Supplementary Fig. S1: Flow cytometry analysis of bacterial coating with tSC from skin mucus. Representative dot plots for two different bacterial strains (*S. warneri* and *Flavobacterium sp.*).

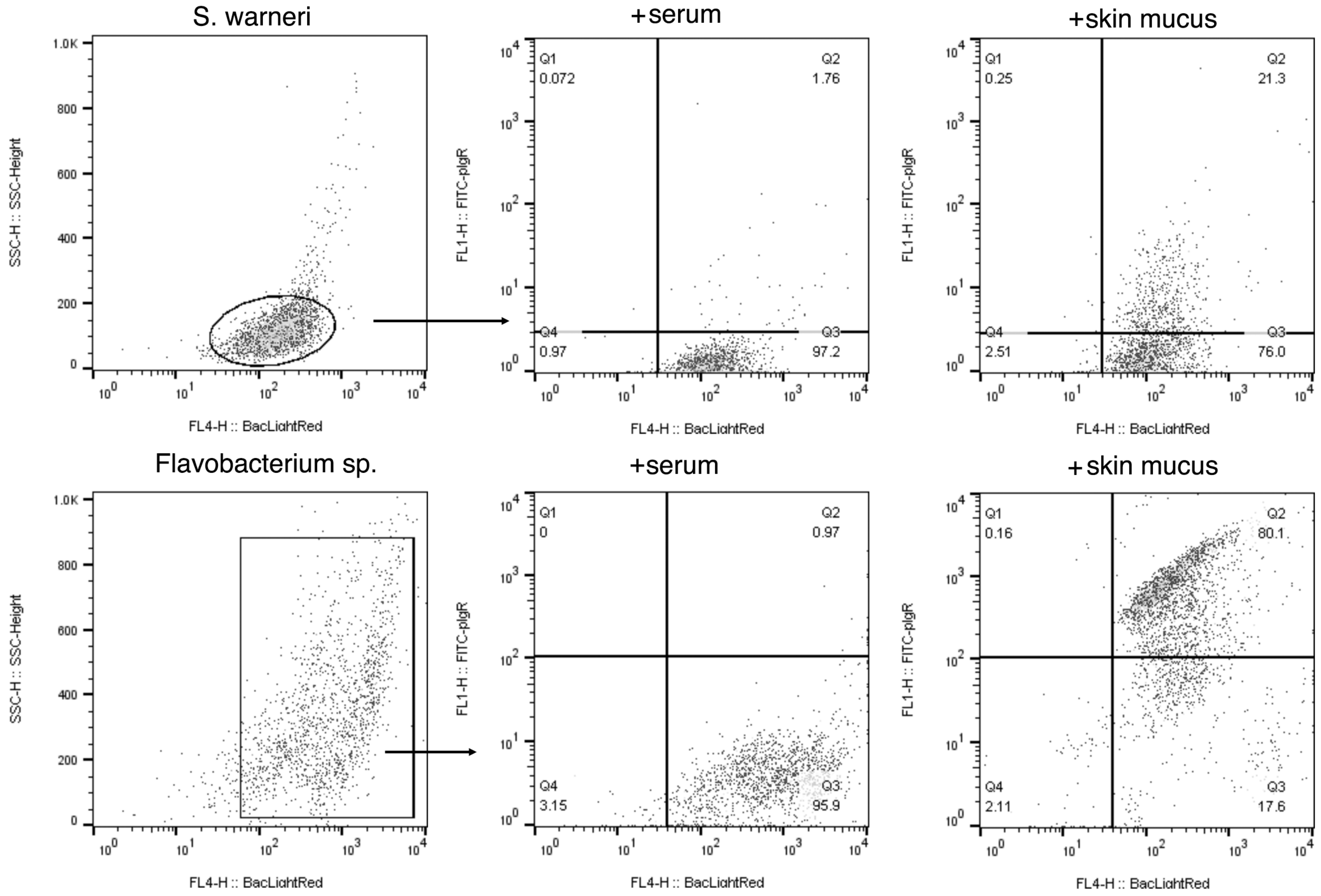

Supplementary Fig. S2: Western blot of rainbow trout gut mucus (G1-G3) and skin mucus samples (S1-S3) showing the presence of trout pIgR. Mucus samples were separated by SDS-PAGE, transferred onto PVDF membranes and probed with rabbit anti-trout pIgR antibody followed by donkey anti-rabbit IgG conjugated with HRP. The detected pIgR bands have the expected molecular weight of 38 KDa and 45 KDa in gut and skin, respectively.

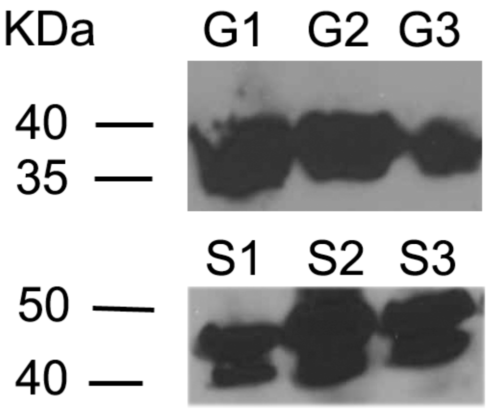

Original blots for Supplementary Fig. S2. Each lane represents a different gut mucus (upper blot) or skin mucus (lower blot) used in the experiments throughout the paper. The same mucus sample was used for all the different bacterial strains to avoid variability due to pIgR concentration in each sample.

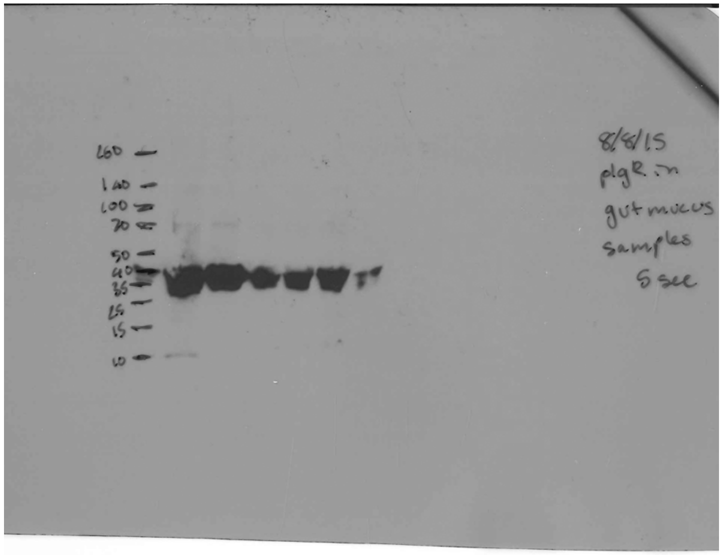

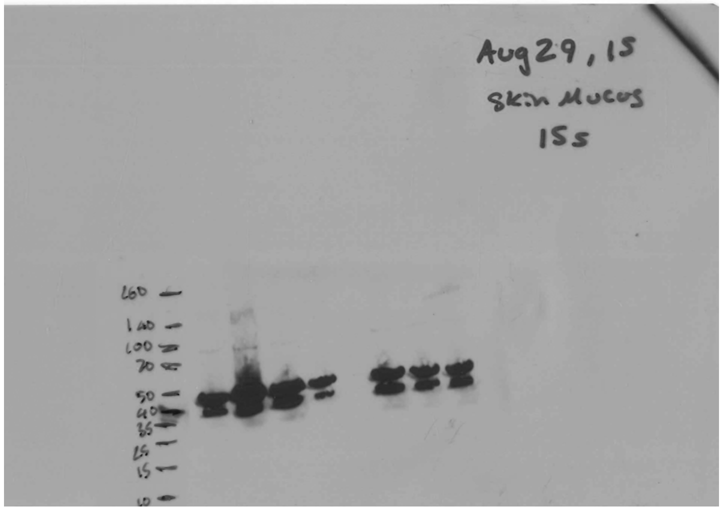

Supplement: Supplementary Information [file srep41753-s1.pdf]
